# Data-Driven Assessment of Wind Turbine Performance Decline with Age and Interpretation Based on Comparative Test Case Analysis

**DOI:** 10.3390/s22093180

**Published:** 2022-04-21

**Authors:** Davide Astolfi, Ravi Pandit, Ludovica Celesti, Matteo Vedovelli, Andrea Lombardi, Ludovico Terzi

**Affiliations:** 1 Department of Engineering, University of Perugia, Via G. Duranti 93, 06125 Perugia, Italy; ludovicacelesti@gmail.com (L.C.); matteo.vedovelli@gmail.com (M.V.); 2 Centre for Life-Cycle Engineering and Management (CLEM), School of Aerospace Transport and Manufacturing, Cranfield University Bedford, Bedford MK43 0AL, UK; raviwithfuture@gmail.com; 3 ENGIE Italia, Via Chiese, 20126 Milano, Italy; andrea.lombardi@engie.com (A.L.); ludovico.terzi@engie.com (L.T.)

**Keywords:** wind energy, wind turbines, technical systems aging, renewable energy, SCADA, data analysis, performance analysis

## Abstract

An increasing amount of wind turbines, especially in Europe, are reaching the end of their expected lifetimes; therefore, long data sets describing their operation are available for scholars to analyze the performance trends. On these grounds, the present work is devoted to test case studies for the evaluation and the interpretation of wind turbine performance decline with age. Two wind farms were studied, featuring widely employed wind turbine models: the former is composed of 6 Senvion MM92 and the latter of 11 Vestas V52 wind turbines, owned by the ENGIE Italia company. SCADA data spanning, respectively, 10 and 7 years were analyzed for the two test cases. The effect of aging on the performance of the test case wind turbines was studied by constructing a data-driven model of appropriate operation curves, selected depending on the working region. For the Senvion MM92, we found that it is questionable to talk about performance aging because there is no evident trend in time: the performance variation year by year is in the order of a few kW and is therefore irrelevant for practical applications. For the Vestas V52 wind turbines, a much wider variability is observed: two wind turbines are affected by a remarkable performance drop, after which the behavior is stable and under-performing with respect to the rest of the wind farm. Particular attention is devoted to the interpretation of the results: the comparative discussion of the two test cases indicates that the observed operation curves are compatible with the hypothesis that the worsening with age of the two under-performing Vestas V52 can be ascribed to the behavior of the hydraulic blade pitch. Furthermore, for both test cases, it is estimated that the gearbox-aging contributes negligibly to the performance decline in time.

## 1. Introduction

The decline with age of technical system performance is an expected phenomenon [1,2,3]. In regards to wind turbines, there are no theoretical standards for the expected rate of performance worsening with age. The only possibility is therefore learning from experience, which means from data. This has gradually become a more timely problem as a growing number of wind turbines have been reaching the end of their expected lifetime: actually, for example, the percentage of wind turbines aged fifteen years or more is in the order of 50% in some European countries as Germany, Denmark and Spain. The decision between the options of extending the lifetime of old wind farms or repowering or decommissioning [4] can be supported by an improvement in the knowledge of how the technical performance of wind turbines declines in time.

The first studies in the literature regarding the aging of wind turbines are based on the analysis of cumulative data of a vast number of wind turbines: the general principle is attempting a regression between the age and the capacity factor of the analyzed fleet and inquiring if there is a declining trend.

In [5], the public data of 282 wind farms sited in the UK are analyzed and the result is that wind turbines decrease their output by 1.6±0.2% per year, which is a noticeable amount. A similar approach is proposed in [6] for Swedish wind farms and the achieved estimate of performance decline with age is lower with respect to [5]. In [7], the cumulative data of 921 wind farms in Germany, spanning the years 2000–2014, have been analyzed. This study is representative of a tendency in the studies about wind turbine aging: the observation that the evolving technology mitigates the performance decline with age. A relevant observation of the study is that, for the considered fleet, in the year 2000, the average wind turbine capacity was 611 kW, while it was 1453 kW in the year 2015. The reported estimate of the average yearly decline rate is 0.63%. In [8], 917 onshore wind farms in the U.S. have been analyzed in their first ten years of operation. From the achieved results, similarly to [7], a distinction between older plants and newer plants can be formulated. Older plants, installed before 2008, are averagely characterized by a production decline with age in the order of −0.5% per year. For newer plants, online after 2008, a lower decline rate (−0.17% per year) is observed. In [9], cumulative data of the offshore UK fleet have been analyzed and the conclusion is that the technical efficiency does not decline with age. On the other hand, in [10], it is observed that the wind turbine fleet in Texas (the largest in the U.S.) has been undergoing performance decline with age and the interpretation is that the path of decline is quite flat for the first years of wind farm operation, but then accelerates as the facilities age. It would be very interesting to inquire if this path occurs irrespective of the evolving technology or not; reasonably, this issue will be addressed in the upcoming few years. In [11], the worsening performance of wind turbines is observed by another point of view, which is the mismatch between the observed annual energy production in Sweden and the estimate formulated in the wind farm design phase.

The above-cited studies employ gross information (cumulative data) from a vast number of wind turbines, in order to highlight statistically meaningful trends. The advantage of this kind of approach is the statistical robustness; the disadvantage is the lack of control on the interpretation of the performance decline with age. In order to individuate actions for counteracting wind turbine aging and improve the prognosis [12], it is therefore necessary to develop methods based on detailed information (SCADA data) [13]. The first efforts are therefore necessarily concentrated on a reduced number of wind turbines (even one): the objective is a deeper interpretation of how the aging of wind turbines manifests [14].

In [15], the power curve analysis based on International Electrotechnical Commission guidelines has been pursued in order to inquire about the performance degradation rate of wind turbines sited inland and seaside; the estimates are shown to be sensibly different (0.1% against 0.3% average decline of the capacity factor). In [16], criteria are proposed for the estimation of wind turbine aging basing on operation data. There is a substantial agreement in the literature about the fact that wind turbine aging is expected to manifest as a power coefficient decrease, sub-component temperatures and a nacelle vibration increase. The studies in [17,18,19] deal with the Vestas V52 wind turbine (which is also investigated in this study); in particular, for one considered test case sited at the Dundalk Institute of Technology, a remarkable performance decline with age is observed in the form of diminished extracted power for the given generator speed. In [20], LiDAR and SCADA data have been employed for the analysis of the performance aging of three Mitsubishi MWT-1000A, having 1 MW of rated power, and the rate of deterioration has been estimated to be −0.52% per year. In [21], a SCADA data analysis is devoted to the formulation of indicators for the aging of the blade pitch system of wind turbines, which are conceptually similar to those in [16]: this kind of study is particularly promising because, as will be highlighted in the present study, the blade pitch is a critical component of the machines.

### Contribution to the Knowledge

The goal of this research is to assess the deterioration in wind turbine performance with age for various technologies and to provide a relevant interpretation for the observed occurrences. This research is a partnership between academia and industry and is based on a discussion of two test cases with widely used wind turbine models: 6 Senvion MM92 in WF1 and 11 Vestas V52 in WF2. These test cases were chosen because the wind turbines in WF1 have a rated power of 2 MW and use electrical pitch control, whereas the wind turbines in WF2 have a rated power of 850 kW and use hydraulic pitch control: thus, these two case studies belong to different wind turbine technologies (in terms of age, size and control) and it can be instructive to compare performance degradation trends. In order to assess the average annual rate of performance degradation with age, dedicated approaches for the analysis of long-term SCADA data were used.

Furthermore, by employing appropriate techniques based on operation curves analysis [18] and wisely interpreting the comparative test case analysis in light of the principles of wind turbine control, it is possible to individuate the sub-components mostly related to the observed performance decline with age.

A remarkable methodological innovation of this study is that the operation region of full aerodynamic load (variable rotational speed and practically fixed blade pitch) has been investigated in depth, with respect to the state-of-the-art in the literature. This study has also inquired to what extent the performance analysis can be pursued through a univariate approach based on the relation between the sole rotational speed and the power.

The following are the main novel aspects of this study:

- In a broad sense, the approach to quantifying wind turbine performance decline with age based on SCADA data analysis;
- The discussion about the meaning of wind turbine performance decline with age, given that, as discussed in Section 4, the collected results indicate that it is questionable to pose that efficiency declines at a certain rate per year and that the degradation behavior occurs homogeneously for the various wind turbines;
- The interpretation of the observed phenomena in relation to the wind turbine technology rather than to the size of the machine (as is mostly presented in the literature);
- The observation that the hydraulic pitch is a critical component, whose health state should be cautiously monitored, because it is likely connected to the performance degradation.

The structure of the manuscript is as follows: at first, the test case and the data sets are briefly described in Section 2; subsequently, the methods are discussed in Section 3; the results are summarized in Section 4; conclusions and further directions are provided in Section 5.

## 2. Test Cases and Data Sets

WF1 is composed of 6 Senvion MM92 wind turbine and WF2 of 11 Vestas V52 wind turbines. These test cases have been selected because the featured wind turbine models are employed worldwide and are therefore representative of 2 MW and <1 MW wind turbines. WF1 and WF2 have been operating respectively since April 2010 and September 2007. Furthermore, another qualifying difference between WF1 and WF2, which motivates the selection of these two test cases, is that in WF1 the wind turbines have electrical pitch control, while in WF2 the pitch control is hydraulic. It should be noticed that WF1 and WF2 employ doubly fed induction generators of the same main manufacturers, which are not reported here for confidentiality issues. The blades of WF1 and WF2 have been inspected periodically by the wind turbine manufacturer. It should be noticed that WF1 has been considered as a test case also in [13] (indicated as WF2 in that study): it has also been selected for this study because it is the test case with electric pitch control for which the authors have at disposal the longest data set.

The available data sets and the main features of the test cases are indicated in Table 1. The data have been divided into yearly packets. Notice that for WF1, we are practically dealing with the first ten years of operation, while the data set for WF2 corresponds to ages seven to fourteen.

The measurements at disposal have 10 min of averaging time, as typical in SCADA data analysis, and are:

- Wind speed *v* measured at the nacelle of each wind turbine [m/s];
- Active power *P* [kW];
- Rotor speed ω [rpm];
- Generator speed Ω [rpm];
- Blade pitch β ${[}^{\circ }]$;
- Run time counter [s].

For each wind turbine, the data are filtered on normal operation by requesting the run time counter to be 600 s out of 600.

In order to take into account as much as possible the effect of environmental conditions, it is recommended to renormalize the nacelle wind speed *v* by considering the effect of air density as indicated in Equations (1) and (2):

(1) $$v_{c}=v{\left(\frac{\rho }{\rho_{ref}}\right)}^{\frac{1}{3}}$$

(2) $$\rho =\rho_{ref}\frac{T_{ref}}{T_{amb}}$$

where $v_{c}$ is the corrected wind speed, *v* is the estimate of undisturbed wind speed provided by the wind turbine nacelle anemometer, ρ is the air density measured on site, $\rho_{ref}=1.225\;\mathrm{kg}/m^{3}$ is the air density in standard conditions, $T_{ref}$ is the absolute temperature in standard conditions (288.15 K) and $T_{amb}$ is the absolute ambient temperature measured on site. Nevertheless, it should be noticed that, also upon renormalization, the measurements from the cup anemometers are affected by critical points [22]: in order to circumvent them as much as possible and to strengthen the consistency of the present analysis, the wind speed measurements are employed only for data filtering in order to discriminate between the operation regions of the wind turbines, as discussed in Section 3.

## 3. Methods

### 3.1. Operation Curves Analysis

**Figure 1:**
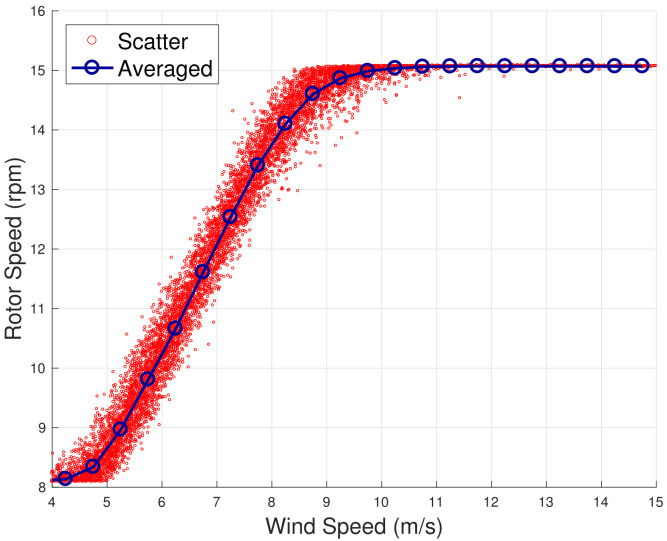
A sample scatter and binned wind speed—rotor speed curve for WF1.

**Figure 2:**
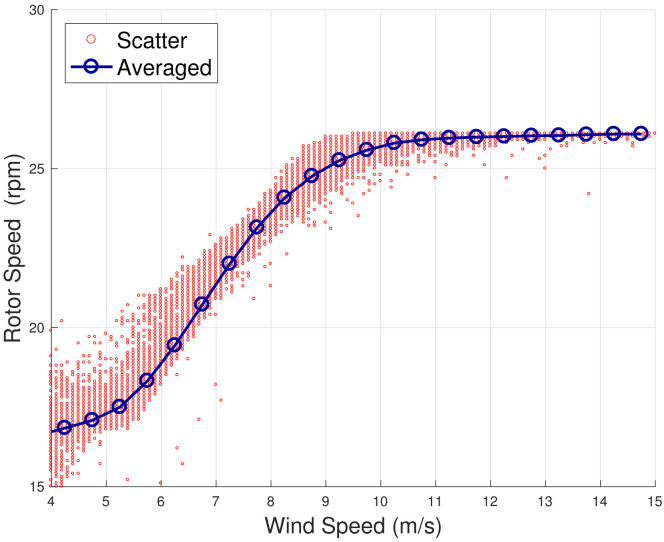
A sample scatter and binned wind speed—rotor speed curve for WF2.

**Figure 3:**
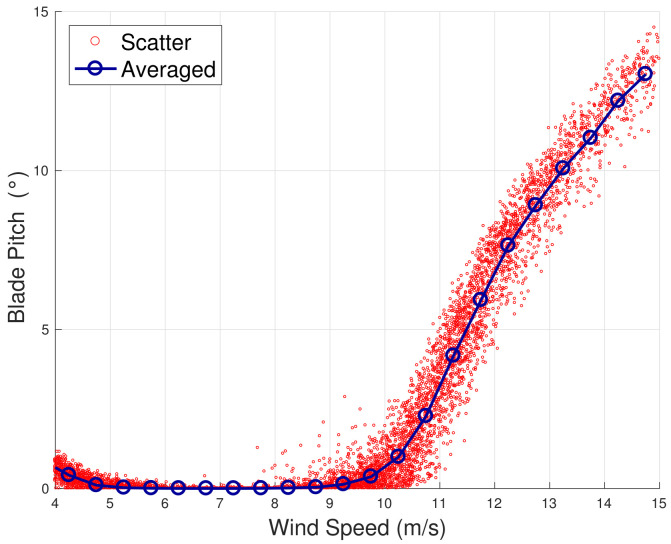
A sample scatter and binned wind speed–blade pitch curve for WF1.

**Figure 4:**
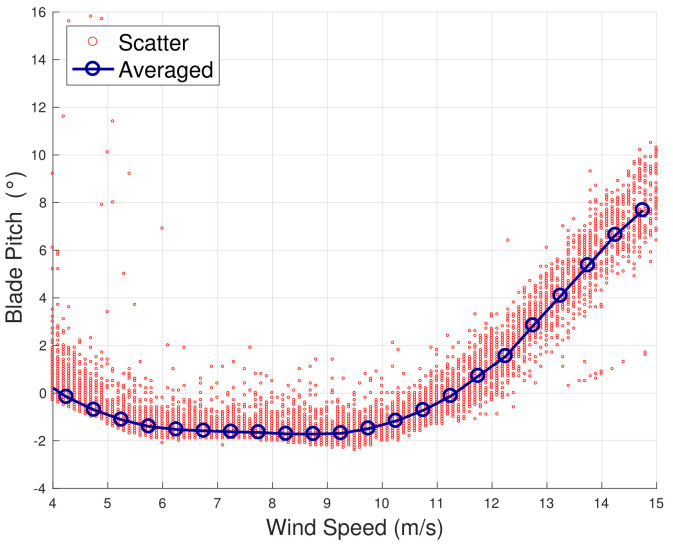
A sample scatter and binned wind speed–blade pitch curve for WF2.

The method is inspired by the analysis in [13,18], which takes into account the principles of the functioning of the wind turbine control. When the wind speed is moderate (approximately 5≤v≤9 m/s), the wind turbine operates at the highest aerodynamic efficiency by regulating the rotational speed and keeping the blade pitch substantially fixed. When the wind speed increases (approximately 9≤v≤13 m/s), the wind turbine operates in partial aerodynamic load, by regulating the blade pitch and keeping the rated rotational speed fixed. In this regard, a nomenclature for the operation regions is introduced in Table 2. This behavior can be appreciated from Figure 1, Figure 2, Figure 3 and Figure 4 for WF1 and WF2. It should be noticed that the features of these figures support the selection of the test cases of the present study. Actually, the behaviors of WF1 and WF2 are similar but display slight differences: the electric blade pitch of WF1 (Figure 3) has less variability with respect to the hydraulic blade pitch of WF2 (Figure 4), in particular, in the full aerodynamic load regime; this in turn is related also to the control of the rotational speed.

Furthermore, as a general motivation for the approach of this study, it should be noticed that the use of the wind turbine cup anemometer data has noticeable critical points (as discussed for example in [22]) and it is therefore valuable to estimate the performance without necessarily using the wind speed measurement.

It should be noticed that the reasoning above is an approximation, because for example, the blade pitch is regulated as well in Region 2 through minor adjustments that keep the wind turbine in the optimal working region. Nevertheless, it is a reasonable approximation that is cited as well in the international standards. For this reason, it is more practical to qualitatively observe the trend of wind turbine behavior by employing curves in a plane, which are rotational speed–power in Region 2 and blade pitch–power in Region 2 12. The visualization of these curves can provide a qualitative indication of a trend but, in order to obtain a quantitative estimate of the aging, a model is needed, as is discussed in the following Section 3.2.

The usefulness of having at hand a model such as the one described in Section 3.2 is that it can be easily generalized to be multivariate; thus, the above considerations regarding the blade pitch and rotor speed control in relation to the power can be explored more in depth.

### 3.2. Support Vector Regression

The principles of the support vector regression are detailed here for completeness: the experienced reader can skip this part. It is instructive to start from a linear model as detailed in Equation (3):

(3) f(X)=Xβ+b,

where X is the matrix of regressors, β is the vector of regression coefficients and *b* is the intercept vector. The objective is to find f(X) with the minimum norm value $\beta^{\prime }\beta$ subject to the condition that the residuals between the measurement *Y* and the model estimate f(X) are lower than a threshold ϵ for each *n*-th observation (Equation (4)):

(4) $$\left|Y_{n}-X_{n}\beta +b_{n}\right|\leq \epsilon$$

The support vector regression is therefore a constrained optimization problem, which can conveniently be rephrased in terms of Lagrange multipliers. The function to minimize is $L\left(\alpha \right)$, given in Equation (5):

(5) $$L\left(\alpha \right)=\frac{1}{2}\sum_{i=1}^{N}\sum_{j=1}^{N}\left(\alpha_{i}-\alpha_{i}^{*}\right)\left(\alpha_{j}-\alpha_{j}^{*}\right)X_{i}^{\prime }X_{j}+\epsilon \sum_{i=1}^{N}\left(\alpha_{i}+\alpha_{i}^{*}\right)+\sum_{i=1}^{N}Y_{i}\left(\alpha_{i}^{*}-\alpha_{i}\right),$$

with the constraints (Equation (6)) on the coefficient α and $\alpha^{*}$ of the regression:

(6) $$\begin{array}{c} \sum_{n=1}^{N}\left(\alpha_{n}-\alpha_{n}^{*}\right)=0\\ 0\leq \alpha_{n}\leq C\\ 0\leq \alpha_{n}^{*}\leq C, \end{array}$$

where *C* is the box constraint.

The solution for the β coefficients is given in Equation (7):

(7) $$\beta =\sum_{n=1}^{N}\left(\alpha_{n}-\alpha_{n}^{*}\right)X_{n}.$$

If either $\alpha_{n}$ or $\alpha_{n}^{*}$ is non-vanishing, the corresponding observation (row of the X matrix) is called a support vector (hence the name of the regression).

Once a model has been trained on a data set composed of input variables X and output *Y*, it can be used for predicting new values, given a new input variables matrix $X^{\prime }$, as indicated in Equation (8):

(8) $$f\left(X\right)=\sum_{n=1}^{N}\left(\alpha_{n}-\alpha_{n}^{*}\right){X^{\prime }}_{n}X+b.$$

A non-linear support vector regression is obtained by replacing the scalar products between the observations matrix with a kernel function (Equation (9)):

(9) $$G\left(X_{1},X_{2}\right)=\langle \varphi \left(X_{1}\right)\varphi \left(X_{2}\right)\rangle ,$$

where φ is the transformation mapping of the X observations into the feature space.

A Gaussian kernel selection is given in Equation (10), where κ is the kernel scale:

(10) $$G\left(X_{i},X_{j}\right)=e^{-\kappa {∥X_{i}-X_{j}∥}^{2}}.$$

Equation (8) allows for predicting new values, given the input, and is rewritten as seen in Equation (11):

(11) $$f\left(X\right)=\sum_{n=1}^{N}\left(\alpha_{n}-\alpha_{n}^{*}\right)G\left(X_{n},X\right)+b.$$

The selection of the support vector regression as the model type is supported by the detailed comparative analysis reported in the previous works [13,23], where appropriate statistical measures are discussed.

### 3.3. Aging Estimate

The regression described in Section 3.2 can be considered a replica of the behavior of the selected wind turbines. The general rationale for employing a similar computational tool is given by the fact that the aging of the wind turbine (or in general a performance change) has to be quantified as the difference between the power measured in a given period and an estimate of how much power the wind turbine would have produced if it behaved, for the same conditions, as in a reference period. Given the fact that wind turbines operate under non-stationary conditions, the latter quantity has to be learned from the reference data; this is the reason why machine learning methods are particularly appropriate.

The data-driven model was employed in this study for the quantitative estimate of the wind turbine performance decline with age through the following steps:

- Select a target data set, i.e., the year of interest;
- Select as reference data set, the year previous to the target;
- Divide the reference data set into a fraction for training (D0) and a fraction for testing (D1). Reasonable proportions are $\frac{2}{3}$ and $\frac{1}{3}$;
- Use D0 for training the model by feeding the input variables and the output to the SVR;
- Use the model (trained as above indicated) for predicting the output in the D1 and D2 data sets by feeding the input to the regression and employing Equation (11);
- Compute the residuals between measurements and model estimates for data sets D1 and D2 (indicated as $R_{1}$ and $R_{2}$);
- Compare $R_{1}$ and $R_{2}$ statistically.

The above steps are general and can be applied to whatever model structure and input variable selection, provided that the output of the regression is the power of the wind turbine of interest. For the objectives of this study, the operation regions were separated as indicated in Table 2 and consequent operation curve selection was performed. The input variables and the target of the regressions are therefore indicated in Table 3 for, respectively, the rotor speed–power, the generator speed–power and the blade pitch–power curves. Furthermore, based on the considerations in Section 3.1, the regression can be generalized to be multivariate: the slight regulation of the blade pitch that is also activated in Region 2 can be taken into account by including the blade pitch as an additional input variable to the model. This line of reasoning could be further generalized by including other covariates that are considered of interest for the analysis of the aging, such as temperature or vibration.

The comparison between the residuals $R_{1}$ and $R_{2}$ and the consequent estimation of aging is as follows [24]. Consider Equation (4) with i=1,2.

(12) $$R_{i}\left(X_{i}\right)=Y\left(X_{i}\right)-f\left(X_{i}\right).$$

For i=1,2, one computes (Equation (13))

(13) $$\Delta_{i}=100\frac{\sum_{X\in Data_{i}}\left(Y(X)-f(X)\right)}{\sum_{X\in Data_{i}}Y\left(X\right)}$$

and the quantity in Equation (14)

(14) $$\Delta =\Delta_{2}-\Delta_{1}$$

provides an estimate of the performance deviation from data set D1 to D2.

It should be noticed that the above method splits the performance analysis in the two main operation regions below the rated power: this is performed because the effect of performance degradation is visible only below rated power. Nevertheless, and also in light of a clearer comparison against other types of results in the literature, it can be interesting to formulate a metric for the performance loss in units of annual energy production (which includes production at rated power). Indicating with *E* the total production for a given year, with $E_{Reg}$ the total production for a given year in the selected region (2 or 2 12) and with Δ the corresponding aging estimate for a given year, it is possible to write Equation (15) for $\Delta_{Reg}$:

(15) $$\Delta_{Reg}=\frac{\Delta \cdot E_{Reg}}{E}.$$

where, therefore, $\Delta_{Reg}$ is the average energy yield loss in the region of interest (2 or 2 12) in proportion to the overall energy yield of the wind turbine in the year. Finally, for each wind turbine, the obtained estimates of $\Delta_{Reg}$ can be averaged on all the yearly data sets at disposal, in order to obtain the average yearly decline rate for each working region.

### 3.4. Interpretation of the Role of the Sub-components

The interpretation of the behavior of the test cases in terms of the sub-components is the most critical and innovative aspect of the present study. The considerations drawn in Section 4 arise from the combination of several analyses:

- The data-driven results about the aging estimate;
- The comparative test case analysis;
- The principles of wind turbine control.

Regarding the data-driven analysis, the regressions indicated in Table 3 provide, in Region 2, two estimates of Δ (Equation (14)) for each target data set, which can be indicated, respectively, as $\Delta_{g}$ and $\Delta_{r}$. It is worth discussing the difference between them: given two data sets (reference and target), $\Delta_{g}$ quantifies the average difference in how much power is extracted for a given generator speed. It is reasonable to perform a simplified position: that the amount of power extracted for a given generator speed depends primarily on the working point of the machine (which depends on the blade pitch), on the efficiency of the generator and on the power conversion for feeding into the grid. Similarly, it can be posed that the amount of power extracted for a given rotor speed depends on the working point of the machine, on the efficiency of the main components placed after the rotor, which are the gearbox and the generator, and on the power conversion. It is therefore reasonable as well to pose Equation (16):

(16) $$\Delta_{r}=\Delta_{g}+\Delta_{gear},$$

where $\Delta_{gear}$ is the estimate of the aging effect due to gearbox, which can be evaluated by the difference from Equation (16). Notice that a further estimate of $\Delta_{g}$ and $\Delta_{r}$ is obtained from the multivariate models, which also include the blade pitch and are indicated in Table 3: these are labeled as $\Delta_{gp}$ and $\Delta_{rp}$, respectively; it is interesting to compare them against $\Delta_{g}$ and $\Delta_{r}$ and inquire as to whether they are compatible.

In Region 2 12, instead, only one estimate of aging is obtained, which can be indicated as $\Delta_{p}$ because the unique varying operation variable is the blade pitch. Based on reasoning similar to [25], it is likely that $\Delta_{p}$ is as well related to $\Delta_{gear}$, because the efficiency of the gearbox affects the power output especially for moderate–high wind speeds. Nevertheless, it should be taken into account that the behavior of the blade pitch is critical in Region 2 12 and in general in that region, as the gearbox substantially operates at the rated speed: thus, it is non-trivial to estimate how $\Delta_{p}$ and $\Delta_{gear}$ are related.

The above considerations, as is discussed in detail in Section 4, can be drawn similarly for all the considered test cases and the achieved results are expected to be quite general.

The critical role of the hydraulic blade pitch can be comprehended by comparing the achieved results for the data-driven regressions and for the operation curve analysis. The comparison can be pursued in general between the wind farms, in order to highlight if regular behaviors occur and can be associated with the features of the employed technology, and at the level of a single wind farm, in order to highlight how the under-performance is related to the main operation variables.

The principles of wind turbine control [26] are taken into account in order to properly interpret the above comparison. As discussed in detail in relation to the results collected in Section 4, the reverse engineering based on SCADA data analysis is particularly appropriate in order to quantify the consequences of a given behavior but is more critical to apply for individuating its root causes. Nevertheless, SCADA data are available at zero additional cost to wind turbine owners and managers and their optimal exploitation can be very useful in order to address possible further measurement campaigns, which can corroborate the malfunctioning diagnosis. Furthermore, as discussed in detail in Section 4, the observed behavior is compatible with the degradation of the blade pitch rate as discussed in [26] and the comparative test case analysis helps in corroborating this hypothesis.

### 3.5. Justification of the Use of the Support Vector Regression

The selection of the SVR regression has been based on previous studies by the authors [13,27], where it has been observed that it is appropriate for the application to wind turbine operation curves, and on a verification for the test cases of interest, which is summarized here on. Other types of approaches are conceivable, but it should be noticed that, given the above general considerations, typically non-trivial statistical methods are, in general, required: for example, in [28], the covariate matching approach is employed for space–time performance comparison of wind turbines.

In the following, the most common error metrics (mean error ME, mean absolute error MAE, root mean square error RMSE) are reported for the regression testing on a sample yearly data set for WF1. Two thirds of the data are employed for training and one third is used for testing and error metrics evaluation. In relation to Table 3, in Table 4, the error metrics are reported for the rotor speed–power regression in Region 2 and Table 5 is devoted to the blade pitch–power regression in Region 2 12. Several regression types are compared, which are:

- Support vector regression with Gaussian or polynomial kernel;
- Gaussian process regression;
- Polynomial regression (third order);
- Regression ensemble based on 100 regression trees using least-squares boosting;
- Regression ensemble based on 100 regression trees using bagging;
- Feedforward neural network.

It is out of the scope of the present study to report details about each of the above regressions: the interested reader might consult textbooks.

From Table 4 and Table 5, it can be seen that the support vector regression with Gaussian kernel has the lowest error metrics and thus has been selected for this study. The SVR linear or the polynomial both provide error metrics that are considerably higher and, for this reason, these kinds of models are considered too simple for the scope of a precision wind turbine performance analysis and are not employed typically in the literature. It should be noticed that the other selected benchmarks have error metrics in the order of those achieved with the SVR with Gaussian kernel: this is expected and reasonable, because they are non-trivial models. Similar results have been obtained for WF2 and are omitted for brevity.

## 4. Results

### 4.1. WF1

**Figure 5:**
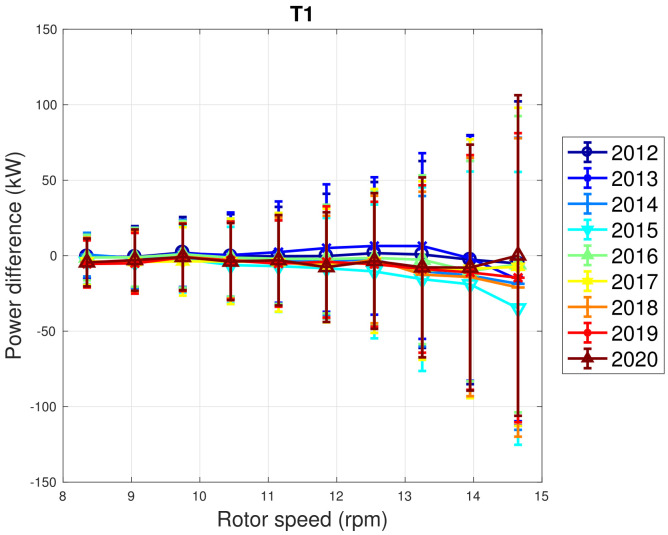
A sample binned rotor speed–power curve in Region 2 for WF1: the curves are reported in the form of difference with respect to the year 2011.

**Figure 6:**
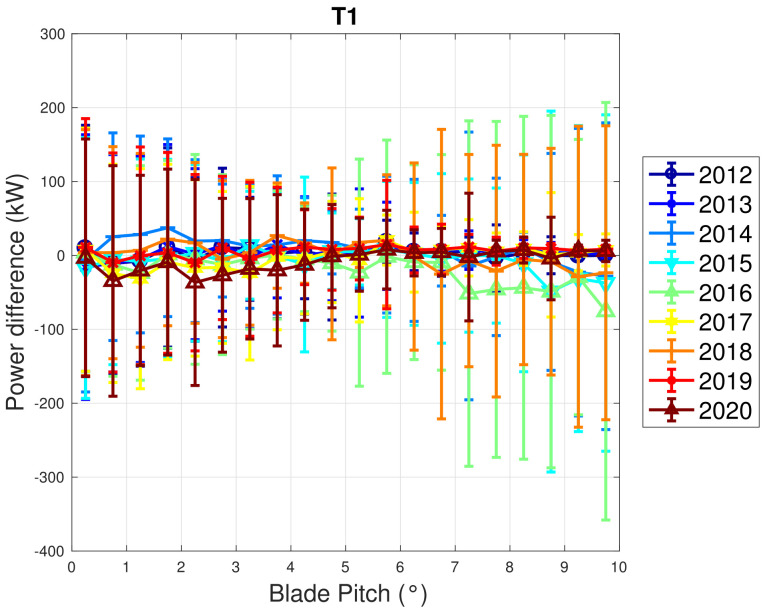
A sample binned blade pitch–power curve in Region 2 12 for WF1: the curves are reported in the form of difference with respect to the year 2011.

In Figure 5 and Figure 6, the trend of the rotor speed–power curve and blade pitch–power curve are appreciable for a sample wind turbine from WF1. The error bars reported in the plots are the standard deviations of the measurements that fall into each bin. It can be seen that most parts of the curves represented in the differences between the rotor curves are negative, which indicates that there is a slight performance decline with respect to the earliest data set. Nevertheless, a clear trend of decline with age does not arise and the reported standard deviation bars indicate that the curves could likely be considered compatible.

The above considerations can be translated in quantitative estimates using the methods of Section 3.2 and Section 3.3, from which the results in Table 6 are obtained for the energy yield decline with age ${\bar{\Delta }}_{Reg}$, averaged on all the data sets at disposal. The first and second columns of Table 6 correspond, respectively, to the estimate labeled $\Delta_{g}$ and $\Delta_{r}$ in Section 3.3: by difference, using Equation (16), it can be argued that the behavior of the gearbox provides a negligible contribution to the performance of the wind turbine. From Table 6, it also arises that the estimates computed with the multivariate regression in Region 2 (thus also including the blade pitch) are practically indistinguishable with respect to those that are based only on the relation between rotational speed and power. By averaging the obtained rates on ten years, the estimates are those reported in Table 6, which noticeably agree with the claims in [8] (−0.17% of decline per year), but would not agree if considering a subset of those 10 years. The lesson coming from SCADA analysis is therefore that cumulative data might yield misleading perceptions of aging and should be treated cautiously.

### 4.2. WF2

**Figure 7:**
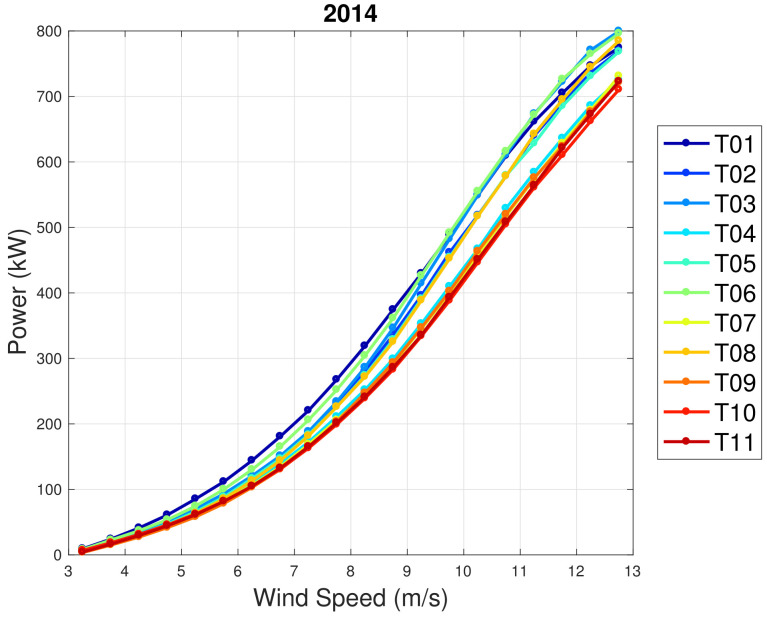
Average power curves for the year 2014: WF2.

**Figure 8:**
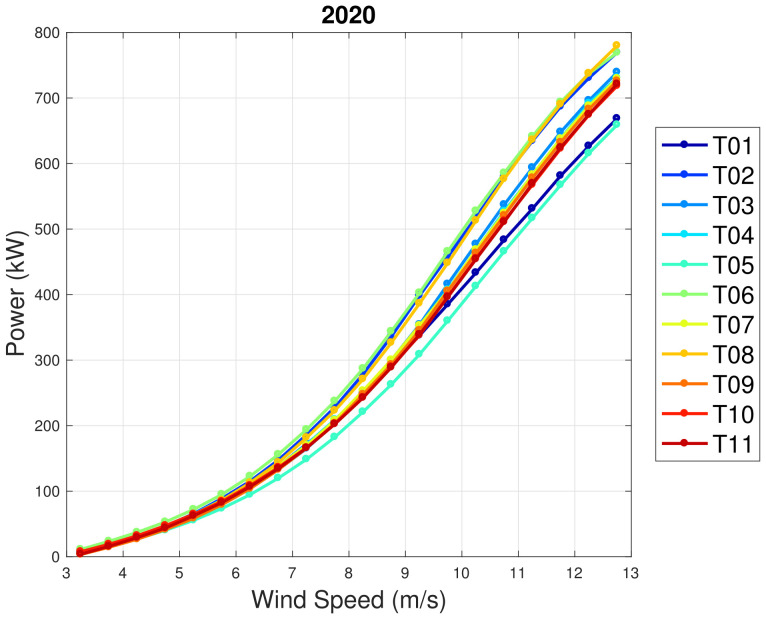
Average power curves for the year 2020: WF2.

In regards to WF2, the general picture of the performance trend with age is much more complex than in WF1. In order to appreciate this, in Figure 7 and Figure 8, the average power curves (computed with the binning method) are reported for the years 2014 and 2020. It arises that T01 was one of the best-performing wind turbines in 2014, while it is the second worst in 2020. Similarly, T05 was in the average of the wind farm in 2014 and it is the worst performing in 2020. According to the line of reasoning in [29], the comprehension of these wind turbines should be prioritized in order to improve the energy yield of the wind farm.

**Figure 9:**
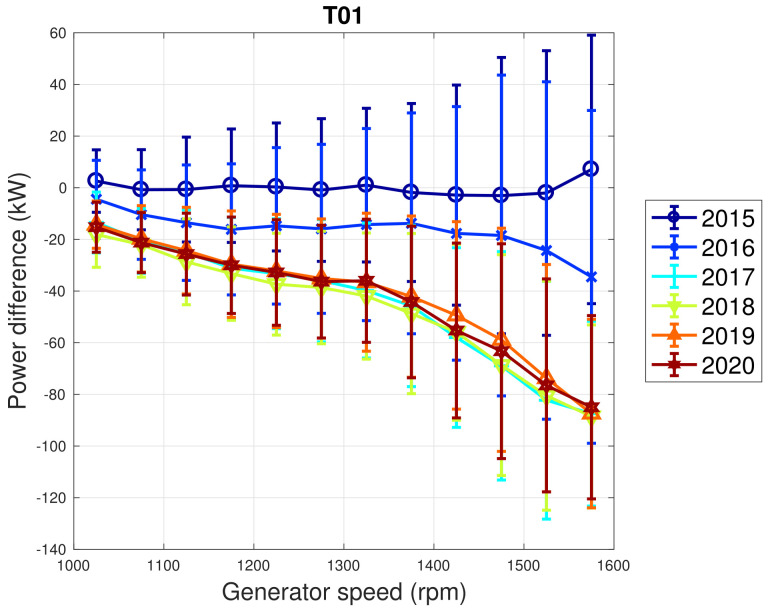
Binned generator speed–power curve in Region 2 for T01 in WF2: the curves are reported in the form of difference with respect to the year 2014.

**Figure 10:**
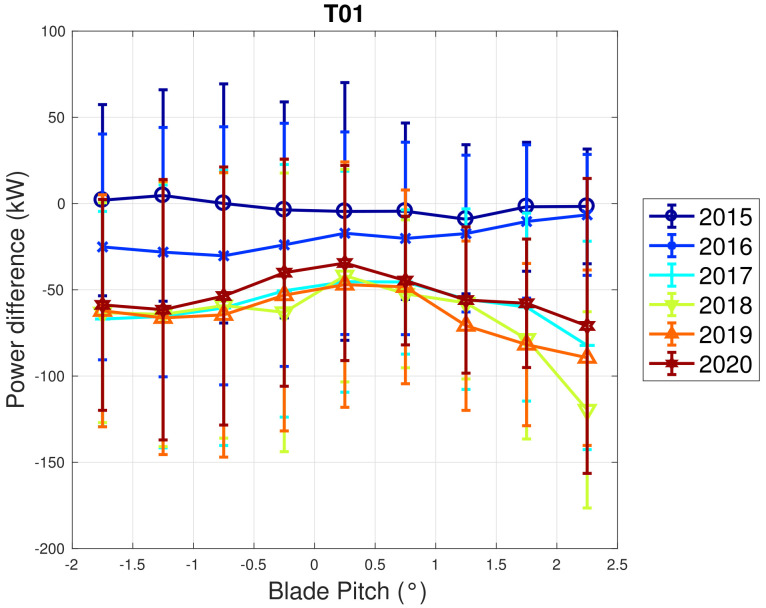
Binned blade pitch–power (right) in Region 2 12 for T01 in WF2: the curves are reported in the form of difference with respect to the year 2014.

**Figure 11:**
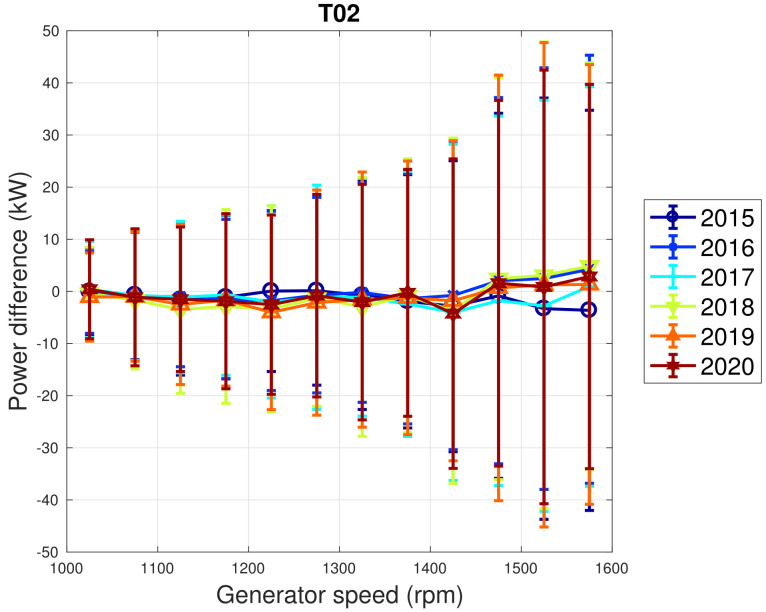
Binned generator speed–power curve in Region 2 for T02 in WF2: the curves are reported in the form of difference with respect to the year 2014.

**Figure 12:**
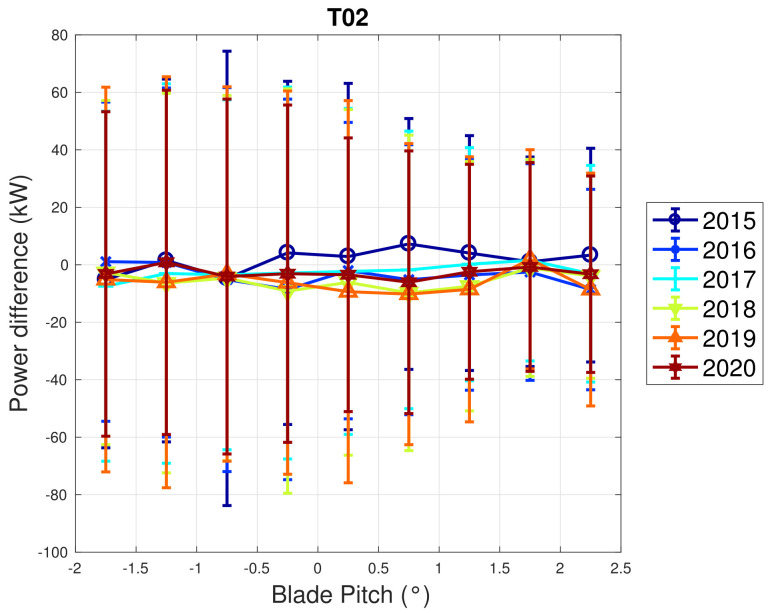
Binned blade pitch–power (right) in Region 2 12 for T02 in WF2: the curves are reported in the form of difference with respect to the year 2014.

Based on the above considerations, in Figure 9 and Figure 10, the trend of the generator speed–power and blade pitch–power curves are reported for T01. Similarly to the case of WF1, indicative error bars are plotted, which are the standard deviations of the power measurements for each rotational speed or blade pitch bin. It arises that the amount of extracted power for a given rotational speed progressively diminished in 2015 and 2016 for T01 and the curves for the year 2017 and subsequent are not compatible with the 2015 curve because the reported error bars do not overlap. An abrupt decline is observed in 2016 and the performance keeps stable from there on. Similar considerations apply to Region 2 12, despite being less in proportion to the performance decrease weights. The same kind of curves are reported in Figure 11 and Figure 12 for T02, from which it arises that the differences in time are only very few kWs and therefore negligible (appreciate the different scale with respect to Figure 9 and Figure 10).

The findings in Figure 9 and Figure 10 raise the question of how to understand what we mean when we say wind turbine aging and what causes it. The behavior of the curves for T01, in particular for the years 2016 and 2017, led to the suspicion that an event occurred in 2016, after which the curves changed and were considerably steady. The so-called $C_{p}-\lambda$ curve, which represents the power coefficient $C_{p}$ as a function of the tip speed ratio λ, is theoretically comparable to Figure 9. The power coefficient is given in Equation (17)

(17) $$C_{p}=\frac{P}{\frac{1}{2}\rho Av^{3}}$$

and in practice it is the ratio between the extracted power *P* and the mechanical power of the wind flow passing through the rotor of area *A*; ρ is the air density and *v* is the wind intensity. The tip–speed ratio λ is defined in Equation (18):

(18) $$\lambda =\frac{\omega R}{v}$$

and is the ratio between the tangential velocity of a point at the tip of the blade (given by the product between rotational speed ω and rotor radius *R*) and the wind intensity *v*.

A decreased extracted power for a given rotational speed (as visible from Figure 9) is equivalent to a decreased power coefficient $C_{p}$ for a given tip–speed ratio λ. Based on the principles of wind turbine functioning, a $C_{p}-\lambda$ curve, which operates at a less-than-optimal working point is achieved by pitching the blades [26]. As discussed in detail in [26], this kind of behavior is compatible with a degradation of the pitch rate control or with a design problem related to the power converter. The latter hypothesis is implausible and has been excluded by the wind turbine manufacturer. Furthermore, the former hypothesis is also corroborated by the comparative test case analysis, because the electrical and hydraulic pitch control have different rates by construction and the latter is more subjected to degradation (being mechanical), while the technology of the converter of WF1 and WF2 is the same.

In fact, coherently with this interpretation, it should also be noticed that the behavior of the blade pitch visibly changes in time for wind turbine T01 (Figure 10), which is different with respect to what happens for T02. In general, it is difficult to use SCADA data analysis for individuating the root cause of a behavior change: in other words, one might ask if a decrease in the power extracted for given rotational speed is the cause of the performance decline or is the consequence of some other problem. The line of reasoning proposed in this study, based on the comparison of multiple test cases with different technology and on the principles of wind turbine control, suggests that in general it is advisable to interpret the data coherently because this can provide meaningful information. In particular, there are two clues regarding the behavior of T01:

- The comparison against WF1 and against the other wind turbines in WF2;
- The behavior of the blade pitch.

**Figure 13:**
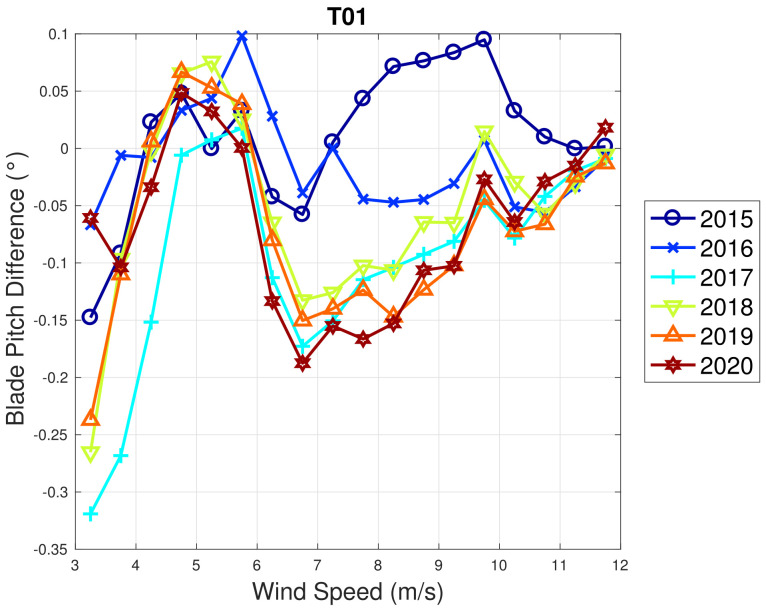
Binned wind speed–blade pitch curve for T01 in WF2: the curves are reported in the form of difference with respect to the year 2014.

**Figure 14:**
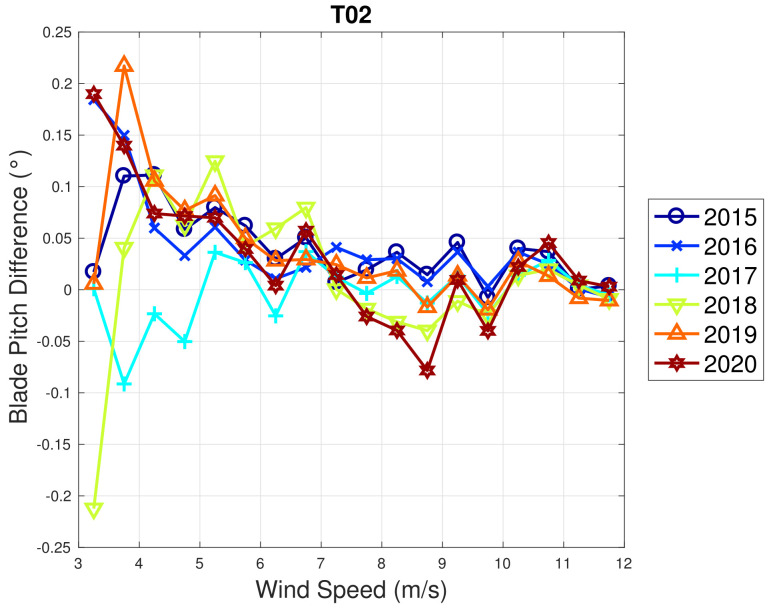
Binned wind speed–blade pitch curve for T02 in WF2: the curves are reported in the form of difference with respect to the year 2014.

**Figure 15:**
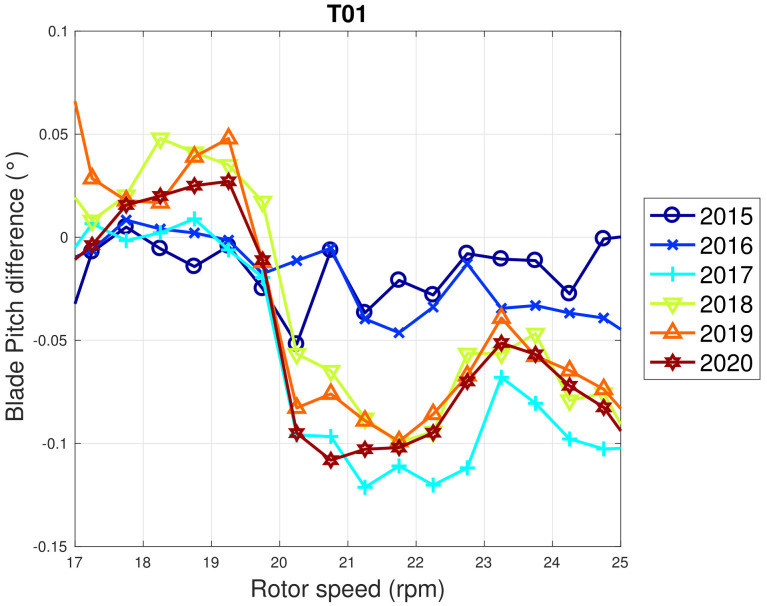
Binned rotor speed–blade pitch curve for T01 in WF2: the curves are reported in the form of difference with respect to the year 2014.

**Figure 16:**
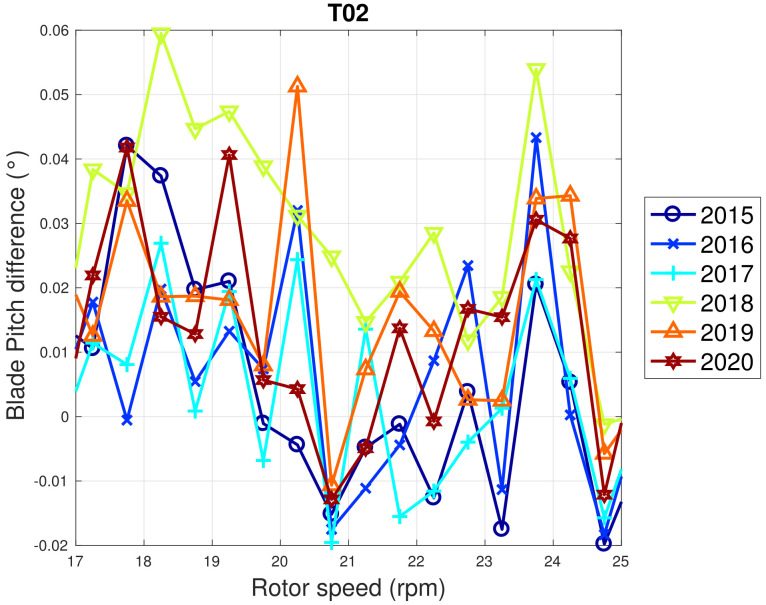
Binned rotor speed–blade pitch curve for T02 in WF2: the curves are reported in the form of difference with respect to the year 2014.

**Figure 17:**
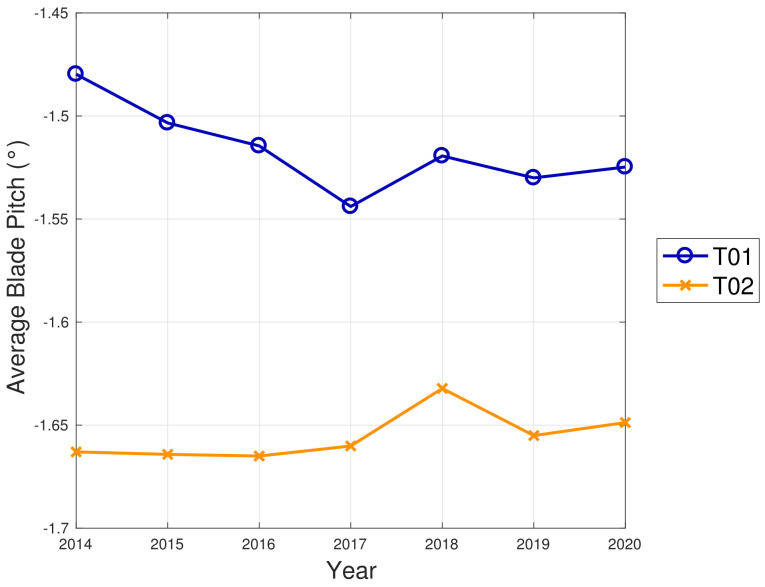
Average blade pitch in region for T01 and T02.

WF1 wind turbines use electric pitch control, but WF2 wind turbines have hydraulic pitch control. Figure 5 compared to Figure 9 is likely related not just to the fact that WF1s wind turbines are newer, but also to the fact that electric pitch control is more efficient (at the cost of a larger failure probability) than hydraulic pitch control (lower failure rate against higher efficiency degradation in time). Figure 13 and Figure 14 illustrate the binned wind speed–blade pitch curves for T01 and T02 in the form of a difference between the curves, which supports this conclusion. Similarly, Figure 15 and Figure 16 report the binned rotor speed–blade pitch curves for T01 and T02 and it arises that they have different shapes and different variability in time. It arises that the curves of T02 change in time considerably less than those of T01, for which instead a sort of shift in the blade pitch settings is observable in 2016 and subsequently since 2017 in a stable manner (as can be seen also from Figure 17). The behavior in Region 2 (according to the nomenclature in Table 2) is particularly worth noticing: in that operation region, the blade pitch on average is held practically fixed. Comparing the year 2015 to the years 2017 and there on, it looks as if the reference blade pitch in Region 2 has undergone at first a major shift and subsequently a further slight drift (see 2018 against 2020). To the best of the authors’ knowledge, such difference in behavior is not due to a different control of T01 with respect to T02: therefore, it is suspected that T01 has undergone a degradation.

**Figure 18:**
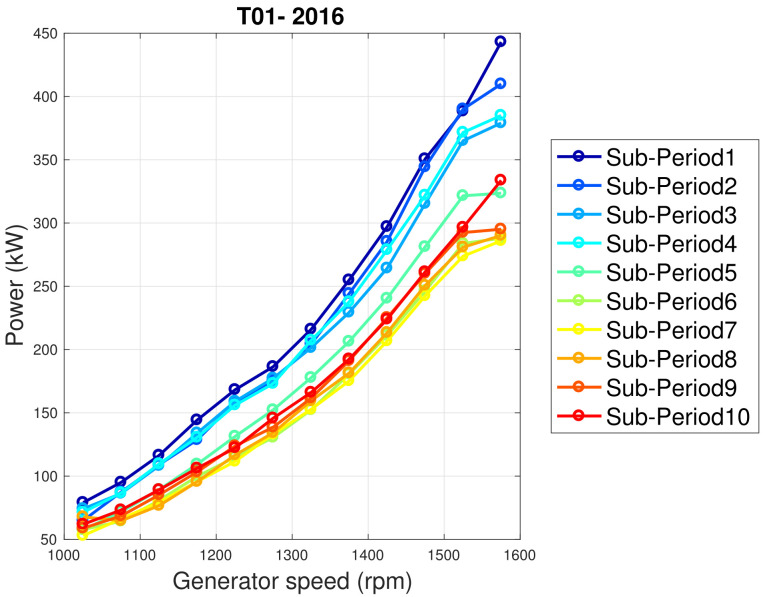
The generator speed–power curve in Region 2 for the T01 wind turbine in WF2 during 2016 (left): data have been divided into 10 sub-periods.

**Figure 19:**
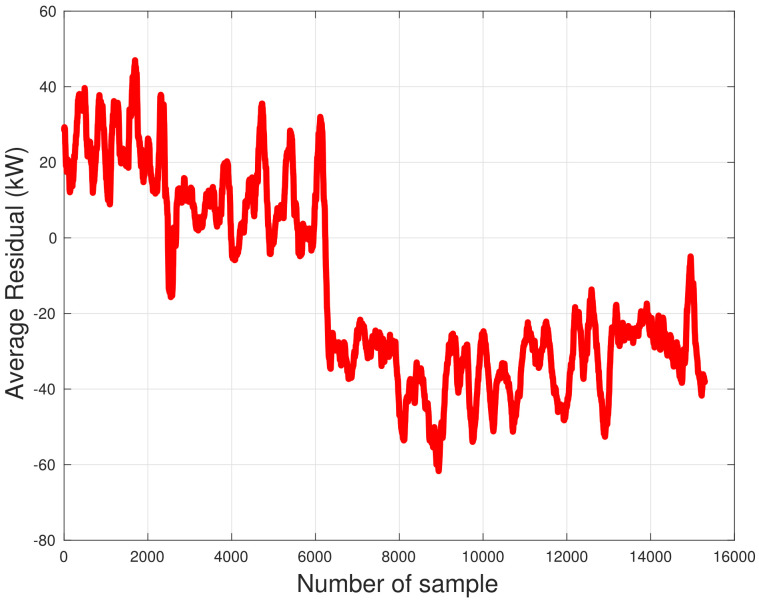
Residual between measurements and model estimates for T01 in WF2 for the year 2016 in Region 2, after being averaged for every 200 datapoints.

A deeper investigation of the SCADA data poses issues also on the time dynamics of the behavior change: in Figure 18, the generator speed–power curve is reported for T01 for the year 2016, upon dividing the data set in 10 sub-periods. Regarding the year 2016, Sub-Period 1 is characterized by the best performance, Sub-Periods 3 and 4 are slightly lower than 1 and 2 and Sub-Period 5 is the bridge with the subsequent periods characterized by worst performance. Based on this observation, the residuals between power measurements of the year 2016 and model estimates (based on the training with the data from the year 2015) have been reported in Figure 19: a moving average composed of 200 data has been employed, in order to smooth the fluctuations. From this figure, it arises that in year 2016, the performance has been degrading progressively and, at a certain point, an abrupt decline occurred, after which the performance has been substantially stable.

The results for the yearly energy yield decline with age, averaged on all the data sets, are reported in Table 7. Remarkable differences between the wind turbines are evident: T01 and T05 are affected by a severe worsening, T03 by a moderate worsening and the other wind turbines substantially, on average, do not change their performance in time. A noticeable result arising from Table 7 is that the $\Delta_{g}$ and $\Delta_{r}$ are indistinguishable, which means, according to Equation (16), that the contribution of the gearbox to the wind turbine performance decline is negligible, in line with what was observed for WF1. Similarly to what happens also for WF1, the estimates of performance decline with age based on the multivariate regression, which means that the blade pitch is indistinguishable with respect to those based on the relation solely between the rotational speed and the power.

## 5. Conclusions

The present study has dealt with the development of SCADA data analysis techniques for the long-term investigation of wind turbine performance and for its interpretation. The objective has been to inquire whether it is possible to individuate clear aging trends and has been motivated by the fact that recent studies in the literature, based on cumulative data analysis, report average yearly rates (order of −0.5% for older wind turbines, −0.2% for newer wind turbines).

Before discussing the main findings of this study, a methodological aspect is worth noting. Based on the principles of wind turbine control, the data analysis has been divided into two regions: the former (called Region 2) is characterized by a practically fixed blade pitch and varying rotational speed; the latter (called Region 2 12) by rated rotational speed and varying blade pitch. In this work, it has been observed that, for the sake of performance analysis, it is definitely acceptable to reduce the relation between rotational speed and power in Region 2. The blade pitch is not exactly fixed in Region 2 but undergoes minor adjustments which maintain the wind turbine in the optimal working point. Nevertheless, in this study, it has been shown that the results of the performance analysis are indistinguishable if one considers the data-driven relation between power (output) and rotational speed and blade pitch (input) or power (output) and rotational speed (input). Evidently, this conclusion is strictly related to the objective, which in this case is long-term performance analysis, and to the type of available data, which in this case are SCADA averaged on a ten minute basis. Other objectives, for example, fault diagnosis, require us to dig more in depth into the dynamic behavior of the machine and therefore the above drawn conclusion would likely be simplistic and the type of data employed in this study would likely be non-optimal.

The results achieved in this study indicate that it is questionable to characterize the performance of wind turbines through average annual yearly decline rates. This conclusion has been developed through the study of two test cases: the former features six Senvion MM92 wind turbines and the latter eleven Vestas V52; respectively, ten and seven years of data have been analyzed.

For the former test case, the observed performance variations are very small and have negligible practical interest. Most of all, there is no clear trend from one year to another.

In regards to the latter test case (Vestas V52 wind turbines), the performance scenario in the wind farm is complex. Most wind turbines are characterized, similarly to the former case, by negligible performance deviations in time. Two wind turbines are instead affected by a performance worsening, which is most clearly visible as diminished extracted power for given rotational speed, whose root cause is critical to determine. The line of reasoning of Section 4 has been formulated based on SCADA-collected evidence, comparative test case analysis and principles of wind turbine control and supports that the observed behavior is compatible with a degradation of the hydraulic blade pitch control: a shift is visible in the wind speed–blade pitch curve also in the operation region where the blade pitch on average is held practically fixed. This should correspond to a less-than-optimal working point, where the $C_{p}-\lambda$ curve is less efficient, which means less extracted power for given rotational speed. As discussed in [26], the capability of controlling the aerodynamic power of a wind turbine is limited by the pitch rate, while the capability of controlling the generator output is limited by the voltage and current rating of the power converter. With a slow pitch rate, additional electrical load may have to be introduced to help slow down or to stall the wind turbine and this results in decreased efficiency. In light of this, the comparative test case analysis selected for this work is particularly instructive because the rate of electrical and hydraulic pitch systems are likely to degrade differently. Given the information available in the SCADA control system of the Vestas V52 wind turbines, the consequences of wind turbine aging are clearly visible but it is more difficult to individuate the causes. Anyway, in general, the aging of the blade pitch system is a very important topic to investigate, as supported by the results in [21], dealing with the relation between blade pitch and wind turbine extracted power, pitch motor efficiency, overheating and vibrations. By this point of view, a fundamental further direction of the present study is the analysis of newer wind turbines with hydraulic pitch control, because it is likely that the SCADA control system provides more measurement channels (as blade piston pressures), which are useful for appreciating a degradation of the pitch rate and an increased stall of the wind turbine. The results of this study therefore support that the efforts for counteracting wind turbine performance decline should be focused on the rotor, through inspections of blade pitch and mass balance, blade pitch actuators degradation and yaw alignment to the wind direction [30].

The average estimates of wind turbine performance aging obtained in the literature with cumulative data should not lead to the wrong expectation that the performance of a wind turbine can be clearly individuated as a declining of a certain rate year by year. The results achieved in this study can be considered quite general, in the sense that it is likely that most wind turbines undergo performance changes that are negligible or at least not distinguishable in the limits of the real-world uncertainties. Unfortunately, a non-negligible fraction of operating wind turbines undergo performance decline in their lifetime: this can happen for a variety of reasons and the decline process can have a variety of characteristic times, which can be clearly highlighted only through appropriate SCADA data analysis. This kind of consideration should be incorporated in intelligent repowering or lifetime extension decisions [4,31,32,33].

Finally, it should be noticed that the computations performed in this study refer to the wind turbines in operation and the periods during which the machines were not productive have been filtered out; therefore, the failure rate is not considered in this study. In a certain sense, it is reasonable to expect that wind turbine aging means increasing failure rate [34] and downtime [35] increasing with time, as discussed also in [21]. This aspect is beyond the scope of this study, but it is an interesting further direction in the wider perspective of a general definition of what one should expect as wind turbines age.

## Figures and Tables

**Table 1:** The available data sets and the test cases.

Wind Farm	Start	End	Rotor Diam. (m.)	Rated Power (kW)	Pitch Control
WF1	1st Jan ’11	31th Dec ’20	92	2000	Electrical
WF2	1st Jan ’14	31th Dec ’20	52	850	Hydraulic

**Table 2:** Wind turbine operation regions considered in this work.

Region	Regime
2	5≤v≤9 m/s
212	9≤v≤13 m/s

**Table 3:** Structure of the SVR regressions for each operation curve.

Region	Curve	Input	Output
2	Generator speed–Power	Ω	Power *P*
2	Rotor speed–Power	ω	Power *P*
2	Generator speed & Blade Pitch–Power	Ω & β	Power *P*
2	Rotor speed & Blade Pitch–Power	ω & β	Power *P*
2 12	Blade pitch–Power	β	Power *P*

**Table 4:** Error metrics for testing of different model types for the rotor speed–power regression in Region 2: WF1, average of the wind farm.

Model	ME (kW)	MAE (kW)	RMSE (kW)
SVR Gaussian	−1.4	23.6	34.5
SVR Polynomial	−2.3	29.6	48.8
SVR Linear	14.5	53.7	70.2
Gaussian Process	2.1	24.6	36.5
Polynomial	3.7	35.6	53.4
Ensemble LS	−2.3	25.1	37.1
Ensemble Bag	−1.9	24.7	36.5
Feedforward ANN	−1.6	23.9	35.7

**Table 5:** Error metrics for testing of different model types for the blade pitch–power regression in Region 2 12: WF1, average of the wind farm.

Model	ME (kW)	MAE (kW)	RMSE (kW)
SVR Gaussian	−2.5	54.6	78.3
SVR Polynomial	−5.6	72.4	94.6
SVR Linear	22.6	103.7	137.2
Gaussian Process	4.8	58.6	76.5
Polynomial	14.7	86.6	101.7
Ensemble LS	−2.8	63.2	79.3
Ensemble Bag	4.2	69.1	81.5
Feedforward ANN	−2.6	57.2	77.4

**Table 6:** Estimates of the average percentage energy yield decline with age for the considered working region and operation curves: WF1. $\Delta_{g}$ and $\Delta_{gp}$ (respectively, $\Delta_{r}$ and $\Delta_{rp}$) quantify the average percentage energy yield change due to the decrease in extracted power for a given generator (respectively rotor) speed in Region 2; $\Delta_{p}$ quantifies the same quantity as a function of the blade pitch in Region 2 12. The metrics are computed according to Equation (15).

Wind Turbine	$\Delta_{g}$	$\Delta_{r}$	$\Delta_{\mathrm{gp}}$	$\Delta_{\mathrm{rp}}$	$\Delta_{p}$
T1	−0.1	−0.1	0.0	0.0	0.06
T2	−0.1	−0.1	0.0	0.0	0.07
T3	−0.2	−0.2	−0.3	−0.3	−0.07
T4	−0.1	−0.1	0.0	0.0	0.05
T5	−0.2	−0.2	−0.2	−0.2	0.02
T6	−0.3	−0.3	−0.3	−0.3	−0.03
Average	−0.15	−0.15	−0.14	−0.14	0.002
Std Dev	0.1	0.1	0.15	0.15	0.002

**Table 7:** Estimates of the average percentage energy yield decline with age for the considered working region and operation curves: WF2. $\Delta_{g}$ and $\Delta_{gp}$ (respectively, $\Delta_{r}$ and $\Delta_{rp}$) quantify the average percentage energy yield change due to the decrease in extracted power for given generator (respectively, rotor) speed in Region 2; $\Delta_{p}$ quantifies the same quantity as a function of the blade pitch in Region 2 12. The metrics are computed according to Equation (15).

Wind Turbine	$\Delta_{g}$	$\Delta_{r}$	$\Delta_{\mathrm{gp}}$	$\Delta_{\mathrm{rp}}$	$\Delta_{p}$
T01	−1.3	−1.3	−1.3	−1.3	−0.5
T02	0.0	0.0	0.0	0.0	0.0
T03	−0.5	−0.5	−0.5	−0.5	−0.2
T04	0.0	0.0	0.0	0.0	−0.1
T05	−0.8	−0.8	−0.8	−0.8	−0.6
T06	−0.2	−0.2	−0.2	−0.2	−0.1
T07	0.1	0.1	0.1	0.1	−0.1
T08	0.0	0.0	0.0	0.0	−0.1
T09	0.0	0.0	0.0	0.0	−0.1
T10	0.1	0.1	0.1	0.1	−0.1
T11	0.0	0.0	0.0	0.0	−0.1
Average	−0.2	−0.2	−0.2	−0.2	−0.2
Std. Dev	0.4	0.4	0.4	0.4	0.2
